# Association of Platelet-Monocyte Ratio with Dyslipidemia in Saudi Arabia: A Large, Population-Based Study

**DOI:** 10.3390/life13081685

**Published:** 2023-08-04

**Authors:** Mohammad A. Alfhili, Ghada A. Alotaibi, Mohammed Alfaifi, Yousef Almoghrabi, Jawaher Alsughayyir

**Affiliations:** 1 Chair of Medical and Molecular Genetics Research, Department of Clinical Laboratory Sciences, College of Applied Medical Sciences, King Saud University, Riyadh 12372, Saudi Arabia; galotaibi2@ksu.edu.sa; 2 Department of Clinical Laboratory Sciences, College of Applied Medical Sciences, King Khalid University, Abha 61421, Saudi Arabia; mhalfaifi@kku.edu.sa; 3 Department of Clinical Biochemistry, Faculty of Medicine, King Abdulaziz University, Jeddah 21589, Saudi Arabia; yalmoghrabi@kau.edu.sa; 4 Department of Research and Development, Al Borg Diagnostics, Jeddah 23523, Saudi Arabia; 5 Department of Clinical Laboratory Sciences, College of Applied Medical Sciences, King Saud University, Riyadh 12372, Saudi Arabia; jalsughayyir@ksu.edu.sa

**Keywords:** dyslipidemia, cardiovascular disease, PMR, biomarker, Saudi Arabia

## Abstract

Background: Abnormal lipid metabolism predisposes to cardiovascular disease. However, dyslipidemia is often asymptomatic leading to its underdiagnosis. Therefore, it is of utmost importance to identify biomarkers that reflect an abnormal lipid profile and trigger the specific investigation of lipid metabolism. The platelet–monocyte ratio (PMR) is a severely understudied index whose association with disturbed lipid markers remains unknown. Methods: A cross-sectional study of the association between PMR and comprehensive lipid profile including total cholesterol (TC), low-density lipoprotein (LDL), high-density lipoprotein (HDL), triglycerides (TG), TC/HDL, LDL/HDL, and TG/HDL in 14,269 Saudi subjects was designed. Prevalence, risk measures, association, and the diagnostic performance (i.e., area under the curve (AUC)) were evaluated. Results: Median PMR was significantly elevated in subjects with high TC (*p* < 0.01), TG, TC/HDL, LDL/HDL, TG/HDL, and LDL and reduced in those with low HDL (all *p* < 0.0001) compared to normal subjects. The increase in PMR was abolished when only males with high TC were considered. Except for TC and LDL, all other abnormal markers were significantly more prevalent when PMR was lower (higher for HDL) than a certain cutoff specific for each parameter. Moreover, the odds of having PMR readings above or below the selected cutoffs are significantly higher with all lipid abnormalities. PMR was also weakly but significantly and differentially correlated with all forms of dyslipidemia (*p* < 0.0001). Notably, the highest diagnostic accuracy of PMR was observed for reduced HDL (AUC = 0.608, *p* < 0.0001) and elevated TG/HDL (AUC = 0.596, *p* < 0.0001). Conclusions: PMR is a novel, inexpensive, and readily available index that is associated with all forms of dyslipidemia, suggesting its potential use in related disorders.

## 1. Introduction

Cardiovascular disease (CVD) constitutes 32% of all mortalities making it the leading cause of death around the world. In Saudi Arabia, the prevalence of coronary heart disease was found to be 5.5%, CVD was 18% in type 2 diabetics, and ischemic heart disease was 32% [1,2]. CVD accounts for 42% of deaths with a projected economic burden of up to $10 billion by 2035 [3]. Dyslipidemia is an established risk factor for CVD with a high prevalence of 32.1–50% in Saudi Arabia, which could be attributed to poor dietary habits and a sedentary lifestyle. Other predisposing factors that are also prevalent in Saudi Arabia include obesity, hypertension, diabetes mellitus, and tobacco smoking [1,4]. In particular, obesity [5] and aging [6] increase the risk for dyslipidemia, which is ameliorated by statins and adoption of a healthy diet and an increased physical activity. In middle- and low-income countries, dyslipidemia had a median prevalence of 43.5% and 63%, respectively [3], with limited accessibility to lipid-lowering drugs [7].

Dyslipidemia significantly contributes to CVD through the development of atheromatous lesions with precipitated cholesterol esters along with platelets, collagen-rich fibrous tissue, and inflammatory cells. In hyperlipidemic conditions, platelets, triggered by low-density lipoprotein (LDL), aggravate the severity of atherosclerosis by releasing inflammatory mediators and recruiting immune cells with ensuing inflammatory and oxidative injury [8]. Platelets overexpress *p*-selectin during atherogenesis, which aids in monocyte recruitment and exacerbates plaque formation through platelet–monocyte aggregation. Circulating monocytes independently predict CVD risk [9], which is attributed to their major role in driving atherogenesis chiefly by forming and propagating fatty streaks due to buildup of cholesterol and other lipids. These cells may also differentiate into dendritic cells, macrophages, and foam cells, which form atherosclerotic plaques. Moreover, immune cell death gives rise to necrotic patches within the plaque complicated by erosion of the fibrous cap and eventual thromboembolism [10].

The platelet–monocyte ratio (PMR) is a novel index of peripheral blood platelet and monocyte counts that is readily available as part of routine laboratory testing. However, there is a dearth of evidence regarding the clinical value of the PMR with very few studies published to date. For instance, it has recently been shown that a PMR of ≤300 was associated with less complete response and reduced survival rates in peripheral T-cell lymphoma patients [11], which indicates its usefulness as a prognostic marker for risk stratification. Furthermore, a PMR of <610 was related to shorter overall survival in retroperitoneal liposarcoma [12]. Similarly, overall survival and event-free survival time in solid childhood tumors were negatively associated with PMR [13]. No reports have thus far examined the association between PMR and dyslipidemia, and the present study therefore aims to determine the prevalence, correlation, risk measures, and diagnostic accuracy of PMR and abnormal lipid markers in a large population.

## 2. Materials and Methods

### 2.1. Study Design

**Figure 1:**
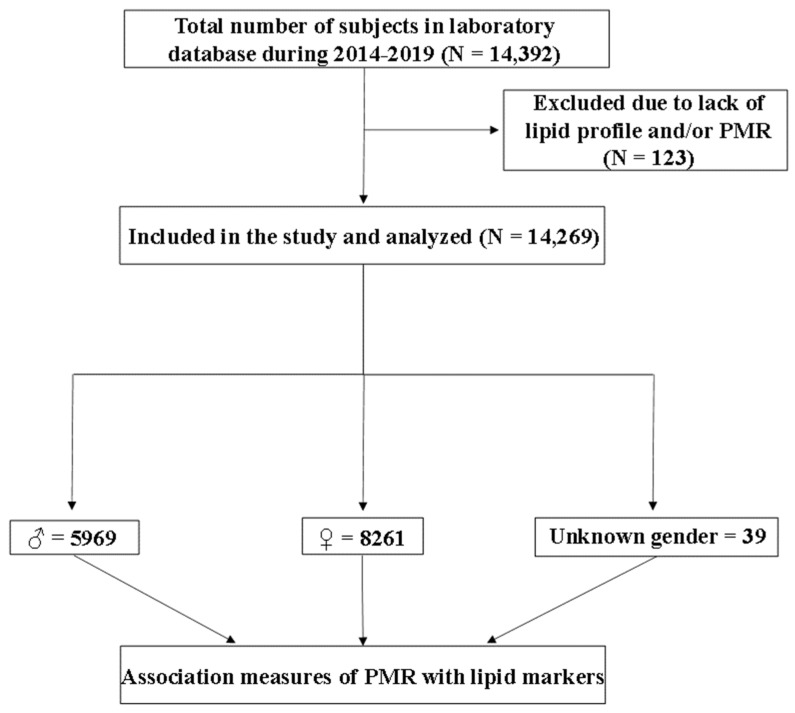
Illustration of study design.

Ethical clearance for this retrospective, cross-sectional study was obtained from Al Borg Diagnostics (IRB approval #07/12). Age, gender, and laboratory records that permitted the calculation of PMR were available for 14,269 subjects aged from 4 to 106 years old (Figure 1). A total of 39 subjects (0.27%) were of unknown age and gender, while 865 (6.06%) were 4–17 years of age, 6707 (47.0%) were 18–39 years of age, 5470 (38.33%) were 40–64 years of age, and 1188 (8.32%) were 65–106 years of age.

Based on receiver operating characteristics (ROC) curve analysis, the highest two area under the curve (AUC) values were observed with HDL and TG/HDL and averaged a cutoff of <588.75, which was considered normal for PMR. Dyslipidemia was confirmed by total cholesterol (TC) of ≥200 mg/dL, LDL of ≥100 mg/dL, high-density lipoprotein (HDL) of <40 mg/dL, triglycerides (TG) of ≥150 mg/dL, a TC/HDL ratio of ≥6, an LDL/HDL ratio of >2.5, or a TG/HDL ratio of >2 [3]. Table 1 shows subject characteristics.

### 2.2. Statistical Analysis

D’Agostino and Pearson test and Kolmogorov–Smirnov test (*p* < 0.0001) indicated that the data were skewed. Therefore, Mann–Whitney U test was used to compare two groups. Results were plotted as medians ± interquartile range (IQR). The prevalence risk (PR) and odds ratio (OR) were calculated (±95% confidence interval; CI) to examine the association between PMR and lipid markers. Decision curves and calibration curves were constructed using SAS v9.3 (SAS Institute Inc., Cary, NC, USA) and R package v4.3.1, respectively. Data were analyzed by GraphPad Prism v9.2.0 (GraphPad Software, Inc., San Diego, CA, USA) with statistical significance defined by a *p* value of <0.05.

## 3. Results

### 3.1. PMR Is Significantly Altered in Distinct Lipid Markers

**Figure 2:**
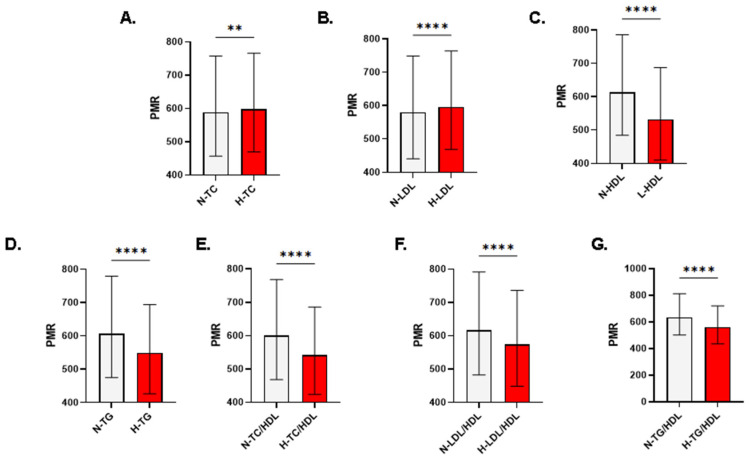
Patterns of PMR in light of lipid markers in both genders. Medians ± IQR of PMR in individuals with normal and abnormal (**A**) TC, (**B**) LDL, (**C**) HDL, (**D**) TG, (**E**) TC/HDL, (**F**) LDL/HDL, and (**G**) TG/HDL. N, normal; H, high; L, low. Significance is indicated by ** *p* < 0.01 and **** *p* < 0.0001.

As shown in Figure 2A, PMR is significantly increased in high compared to normal TC group (588.6 ± 456.9–757.3 vs. 597.9 ± 469.6–765.8, *p* = 0.002). Likewise, in Figure 2B, individuals with high LDL had a significantly increased PMR in comparison to those with normal LDL (580.6 ± 440–748.6 vs. 596 ± 468.1–763.9, *p* < 0.0001). In contrast, PMR was significantly reduced in all other forms of dyslipidemia including low HDL (613.2 ± 484.1–785.7 vs. 531.8 ± 409.4–687, *p* < 0.0001; Figure 2C), high TG (606.1 ± 475–778.9 vs. 549 ± 426.1–694.2, *p* < 0.0001; Figure 2D), TC/HDL (600 ± 468–768.1 vs. 542.3 ± 423.8–685.7, *p* < 0.0001; Figure 2E), LDL/HDL (616.7 ± 482.5–791.6 vs. 573.8 ± 448.3–735.9, *p* < 0.0001; Figure 2F), and TG/HDL (634.5 ± 501.9–811.1 vs. 561.7 ± 435.3–720.5, *p* < 0.0001; Figure 2G).

### 3.2. PMR Exhibits Gender Disparity in TC

**Figure 3:**
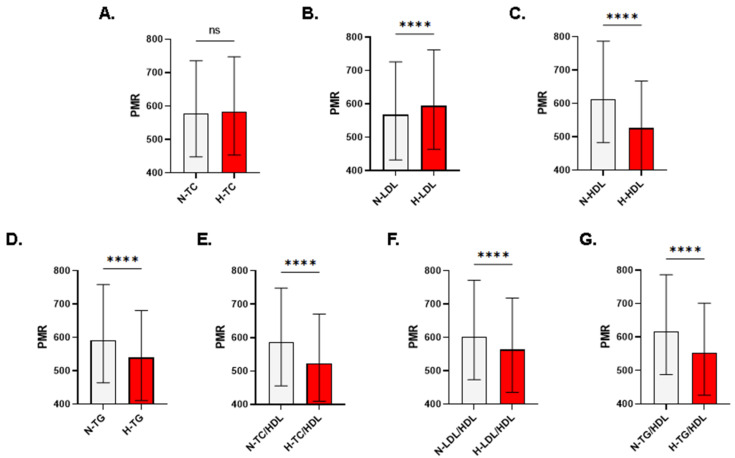
Patterns of PMR in light of lipid markers in males. Medians ± IQR of PMR in males with normal and abnormal (**A**) TC, (**B**) LDL, (**C**) HDL, (**D**) TG, (**E**) TC/HDL, (**F**) LDL/HDL, and (**G**) TG/HDL. N, normal; H, high; L, low. Significance is indicated by ns (i.e., not significant) and **** *p* < 0.0001.

**Figure 4:**
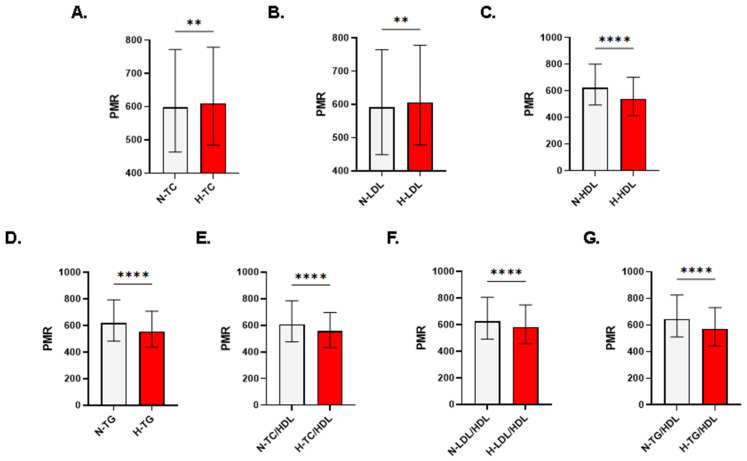
Patterns of PMR in light of lipid markers in females. Medians ± IQR of PMR in females with normal and abnormal (**A**) TC, (**B**) LDL, (**C**) HDL, (**D**) TG, (**E**) TC/HDL, (**F**) LDL/HDL, and (**G**) TG/HDL. N, normal; H, high; L, low. Significance is indicated by ** *p* < 0.01 and **** *p* < 0.0001.

In males, PMR was not significantly different in subjects with normal and high TC (578.4 ± 447.8–735.6 vs. 583.6 ± 453–747.8, *p* = 0.1636; Figure 3A). Except being significantly elevated in subjects with high LDL (Figure 3B), all other forms of lipid disturbances displayed significantly reduced PMR values (Figure 3C–G). In females, the significant elevation in PMR was restored as depicted in Figure 4A (597.6 ± 463.7–772.3 vs. 610 ± 484.4–778.6, *p* < 0.0038). Similar to what is observed when both genders or when only males were analyzed, female subjects also had significantly increased PMR in the high LDL group (Figure 4B) and significantly lower PMR in all other forms of dyslipidemia (Figure 4C–G).

### 3.3. Disturbed PMR Is a Marker of Dyslipidemia

To measure the prevalence of various forms of dyslipidemia relative to PMR, a cutoff PMR value that permits the highest sensitivity and specificity to detect abnormal lipid parameters was chosen. Table 2 shows that decreased HDL was significantly more prevalent in individuals with a PMR >586.4 (*p* < 0.0001). Moreover, elevated TG, TC/HDL, LDL/HDL, and TG/HDL were significantly more prevalent when PMR was <569.3, <563.9, <596.9, and <591.1 (all *p* < 0.0001), respectively.

Accordingly in Table 3, the PR for each lipid disturbance indicates that the proportion of subjects with high TC and LDL and low HDL is 1.04- (*p* = 0.0245), 1.02- (*p* = 0.0033), and 1.57-fold (*p* < 0.0001) greater when PMR was below (or above for HDL) the designated cutoff. Furthermore, the proportion of those with elevated TG, TC/HDL, LDL/HDL, and TG/HDL is significantly less by 0.70, 0.66, 0.86, and 0.78 (all *p* < 0.0001) if PMR was higher than the identified cutoff. In congruence with the prevalence findings, the calculated OR values suggest that the odds of having PMR readings above or below the selected cutoffs are significantly higher when lipid abnormalities are present.

### 3.4. PMR Is Differentially Correlated with Lipid Markers

**Figure 5:**
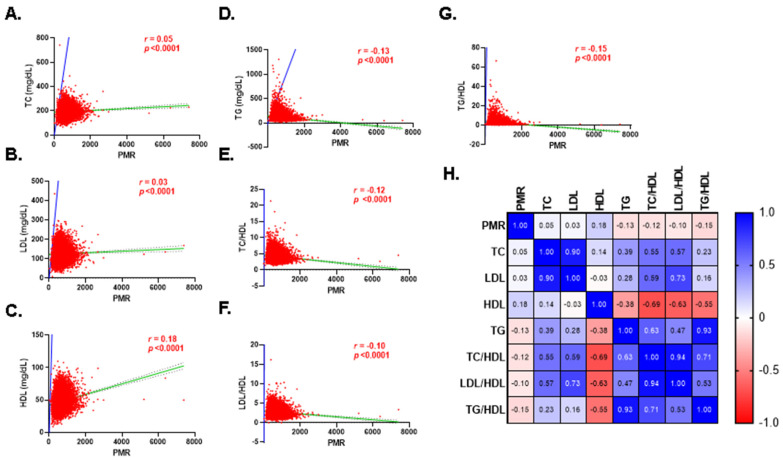
Association of PMR with lipid markers. Spearman’s rank correlation between PMR and (**A**) TC, (**B**) LDL, (**C**) HDL, (**D**) TG, (**E**) TC/HDL, (**F**) LDL/HDL, and (**G**) TG/HDL. Regression lines are shown in green, while identity lines are in blue. A correlation matrix with correlation coefficients is also shown (**H**).

Pearson correlation revealed weak albeit significant positive and negative correlations between all lipid markers and PMR as shown in Figure 5A–H, which points to a possible moderator variable that explains the observed relationship.

### 3.5. Diagnostic Performance of PMR for Lipid Disturbances

**Figure 6:**
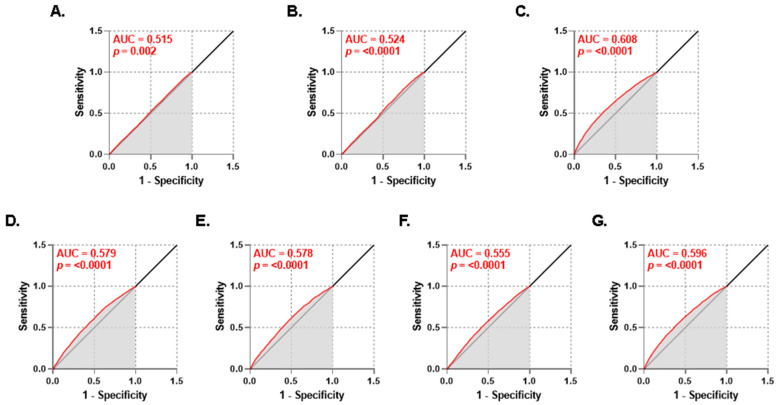
Diagnostic accuracy of PMR. ROC curves along with AUC and *p* values for (**A**) TC, (**B**) LDL, (**C**) HDL, (**D**) TG, (**E**) TC/HDL, (**F**) LDL/HDL, and (**G**) TG/HDL.

We performed a ROC curve analysis in order to evaluate the clinical utility of PMR as a diagnostic tool to discriminate individuals with normal lipid markers and those with dyslipidemia. Figure 6 indicates that the highest AUC was 0.608 (*p* < 0.0001), which was obtained for HDL (Figure 6C) followed by that for TG/HDL at 0.596 (*p* < 0.0001) as shown in Figure 6G. Of note, the very small *p* values provide evidence that the PMR had the ability to distinguish between subjects with normal and abnormal lipid markers.

### 3.6. Predictive Power of PMR for Abnormal Lipid Markers

**Figure 7:**
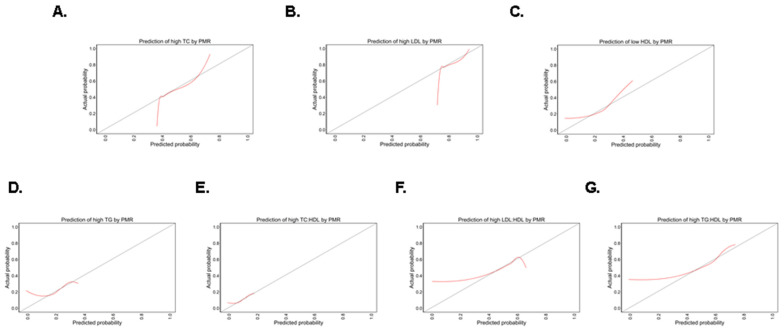
Predictive power of PMR. Calibration curves of PMR for (**A**) TC, (**B**) LDL, (**C**) HDL, (**D**) TG, (**E**) TC/HDL, (**F**) LDL/HDL, and (**G**) TG/HDL.

PMR would detect 40–45% of individuals at a moderate risk to have high TC (Figure 7A), 75–80% of subjects at a high risk to have elevated LDL (Figure 7B), 18–30% of those at a low risk to have decreased HDL (Figure 7C), 18–25% of people at a low risk to have high TG (Figure 7D), 5–18% of subjects at a low risk to have an increased TC/HDL ratio (Figure 7E), 45–60% of individuals at a moderate risk to have an increased LDL/HDL ratio (Figure 7F), and 45–60% of those who are at a moderate risk to have an elevated TG/HDL ratio (Figure 7G). The calibration curves also revealed distinct patterns in which PMR overestimates and underestimates abnormal lipid markers.

**Figure 8:**
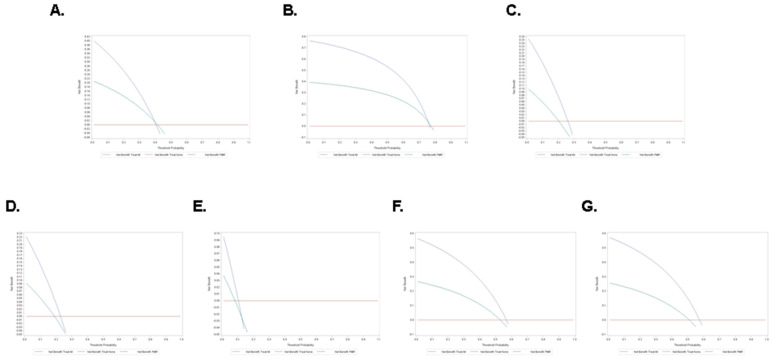
Net benefit of PMR. Decision curves of PMR for (**A**) TC, (**B**) LDL, (**C**) HDL, (**D**) TG, (**E**) TC/HDL, (**F**) LDL/HDL, and (**G**) TG/HDL.

The clinical net benefit and threshold probability of PMR were 0.21 and 0.47 for treating elevated TC (Figure 8A), 0.4 and 0.8 for treating elevated LDL (Figure 8B), 0.1 and 0.27 for treating low HDL (Figure 8C), 0.09 and 0.26 for treating high TG (Figure 8D), 0.38 and 0.17 for treating high TC/HDL (Figure 8E), 0.28 and 0.58 for treating high LDL/HDL (Figure 8F), and 0.26 and 0.55 for treating high TG/HDL (Figure 8G).

## 4. Discussion

Besides being an independent risk factor for CVD, dyslipidemia is also associated with diabetes mellitus, hypertension, anemia, and obesity [3]. Detection of hyperlipidemia is complicated by its silent nature [14], making the early differential diagnosis of underlying conditions challenging [7]. Thus, identifying novel biomarkers to improve screening for risk factors of associated diseases is of great importance. In this retrospective study, we investigated the clinical significance of implementing PMR as an inflammatory marker for the detection of dyslipidemia among Saudi subjects.

Findings of this large-scale, population-based study suggest that most dyslipidemia forms are associated with decreased PMR (Figure 2C–G), pointing at diminished platelets, elevated monocytes, or both. This is in contrast to what is observed when either TC or LDL was increased, in which case PMR was significantly elevated (Figure 2A,B). Alterations in the numbers and functions of circulating platelets and monocytes reflect the critical pathological role of platelet recruitment and monocyte/macrophage infiltration in forming atherosclerotic plaques. Intriguingly, monocyte trafficking but not recruitment seems to be essential for atherosclerosis [15] cementing the importance of monocyte interactions with lipids described in the literature. For instance, HDL has been shown to dampen the inflammatory response through CD11b on monocytes [16], which also engulf oxidized lipoproteins that stimulate their proliferation [9]. Likewise, myeloperoxidase secreted from monocytes builds up in the subintimal space leading to the formation of oxidized lipoproteins and phospholipids, which are ligands for CD36 and promote chemotaxis of monocytes and other immune cells, thereby increasing the atherogenic risk [17].

Singh et al. have recently reported that mean platelet volume, platelet distribution width, platelet–large cell ratio, and platelet aggregation were significantly higher in hyperlipidemics compared to control subjects [14]. In fact, increased platelet size is also seen in coronary artery disease and myocardial and cerebral infarction. Since platelet size correlates with metabolic activity, larger cells are more likely to form a thrombus. Moreover, lipids can modify platelet morphology, aggregation, and degranulation, which impacts other cells including leukocytes and the endothelium [18]. Arterial occlusion due to platelet aggregation is instigated by oxidized LDL and is considered a central mechanism responsible for the worsened prognosis in CVD patients [19].

The underlying mechanism of the sex-based disparity in the association of PMR with serum TC and LDL (Figure 3A and Figure 4A) remains largely elusive. We have recently reported differences in male and female subjects regarding vitamin D regulation [20], which is associated with dyslipidemia [21]. In fact, vitamin D deficiency was positively correlated with the prevalence of dyslipidemia with a significant association observed in males [22]. In contrast, a large study conducted from 2007–2018 indicated that women achieved worse lipid control compared to men [23] because of less compliance with statin therapy due to an increased risk of potential side effects. The gender disparity reported in the current study and in previous reports could be explained at least in part by fat distribution in both sexes. Males have been found to be at a higher risk of developing dyslipidemia compared to their female peers since males tend to accumulate more visceral fat [24]. The relatively increased risk to develop dyslipidemia in men was further corroborated in multiple reports and was variously attributed to occupational stress, unhealthy dietary habits leading to fat accumulation, and a tendency for tobacco smoking and alcohol consumption [25,26,27]. Notably, adiponectin and leptin levels are significantly higher in females, which offers cardiovascular protective properties due to their role in lipid regulation [28,29]. Therefore, the interplay among PMR, bile acids, adiponectin, and leptin, and their individual and overall influence on lipid metabolism deserves further exploration.

The observed gender disparity could also be linked to sex steroids suggesting an alternative mechanism of lipid regulation especially considering the significant role of endogenous estrogen in modulating lipid and carbohydrate metabolism [30]. In particular, estrogens play a significant regulatory role in lipid metabolism via bile acid synthesis as dictated by cholesterol 7α-hydroxylase activity [31]. Since this enzyme is regulated by triiodothyronine [32], the differential effects of thyroid hormones on lipid metabolism in males and females cannot be overlooked. Increased estrogen during pregnancy has been correlated with cholestasis due to compromised bile acid synthesis and hepatic transport [33]. Furthermore, half of breast cancer patients administered tamoxifen as an estrogen receptor antagonist developed hepatic steatosis within two years of therapy [34]. Nevertheless, both exogenous and endogenous estrogen show gender disparity in hepatic dysfunction and are speculated to prevent CVD at physiological concentrations in females [33,35].

Our study revealed that the prevalence rate of dyslipidemia was negatively associated with an elevated PMR, except for those with low HDL (Table 2). The increased proportion of subjects with low HDL when PMR is >586.4 could be attributed, at least in part, to its physiological anti-atherogenic properties by scavenging cholesterol and preventing the development of atherosclerotic lesions [36] and thus lowering CVD risk. HDL also has a direct anti-thrombotic activity as it maintains platelet count and activation via prostacyclin synthesis [37]. Therefore, in addition to its primary role in cholesterol regulation, insufficient or dysfunctional HDL could have detrimental effects on platelet homeostasis and predispose to thrombotic episodes.

Likewise, a reduced PMR in low HDL (Figure 2C, Figure 3C, and Figure 4C) could be beneficial for predicting the potential risk of having CVD. This was corroborated by the fact that individuals with a PMR of >586.4 are 1.8514 times more likely to have diminished HDL (Table 3). Additionally, HDL also inhibits cytokine-induced expression of endothelial cell adhesion molecules thereby minimizing vascular infiltration and inflammation [38]. This was demonstrated in a study reporting that HDL dose-dependently reduced monocyte–endothelium adhesion by targeting CD11b. Contrarily, our study highlighted that those with a low PMR are at risk of having the remaining forms of dyslipidemia, implying an elevated monocyte count, diminished platelets, or both. Along those lines, it has been reported that hyperlipidemia leads to platelet hyperactivity. In particular, oxidized LDL particles cause activation and morphological changes in platelets by stimulating CD36-Src interaction and RhoA/ROCK activation leading to phosphorylation of myosin light chains [19].

Additionally, our study indicates that all lipid markers are significantly but weakly correlated with PMR (Figure 5), suggesting the involvement of a mediator variable. A recent cross-sectional study showed that subjects with increased serum levels of branched-chain amino acids have lower HDL and higher LDL, TG, and lipoprotein insulin resistance score [39]. Furthermore, interactions between lipid species and various mediators related to monocytes and platelets, including resistin [40] and integrins [41], have been described in the literature. Of note, we have recently reported that the monocyte–lymphocyte ratio [42] and polyunsaturated fatty acids [43] are differentially regulated in hyperglycemia, which may be related to changes in PMR seen in hyperlipidemia. However, identification of the nature and role of intermediate variables mediating the correlation between PMR and deranged lipid profile remains to be determined.

Our results demonstrated that the best diagnostic performance by PMR was observed for decreased HDL (Figure 6C) and increased TG/HDL (Figure 6G). An emerging biomarker, TG/HDL has been significantly correlated with insulin resistance and other cardiometabolic conditions including metabolic syndrome and diabetes mellitus [44,45]. In one report, a TG/HDL value of >1.65 in women and >2.75 in men were predictive of metabolic syndrome and first coronary event irrespective of BMI [44]. Nonetheless, the implication of TG/HDL as a biomarker for CVD is limited by the need for a universal cut-off value. Since PMR has demonstrated a superior diagnostic utility in discriminating subjects with elevated TG/HDL, it could thus serve as a promising biomarker in conjunction with TG/HDL for monitoring and predicting the incidence and progression of CVD. Notably, PMR was also successful in predicting individuals with a moderate to high risk of having elevated TC (Figure 7A) or LDL (Figure 7B) as well as the need for treatment of both forms of dyslipidemia (Figure 8A,B). In particular, we found that PMR differentially predicts various forms of abnormal lipid metabolism in low- (reduced HDL and increased TG and TC/HDL), moderate- (elevated TC, LDL/HDL, and TG/HDL), and high-risk groups (elevated LDL). In clinical practice, statins are prescribed for hyperlipidemia and for primary prevention of atherosclerotic CVD. However, major side effects include digestive, neuromuscular, and immune-related symptoms [46]. Moreover, low platelet count and activity have been observed in a subset of patients [47], which warrants cautious utilization and interpretation of PMR before, during, or after commencement of statin therapy. Thus, future studies must be designed to investigate whether PMR precedes, coincides, or follows the onset of dyslipidemia, along with identification of the clinical scenarios in which PMR measurement provides maximum benefit.

Strengths of this study include the very large sample size, minimal analytical variability due to automated data collection and analysis, and the study of a comprehensive lipid panel. Limitations are related to insufficiency of data related to anthropometric measurements such as the body mass index, demographic variables, including socioeconomic status, and lifestyle habits, genetic susceptibility, family history of disease, existing co-morbidities, and current or past medication intake. Furthermore, causal inference could not be made due to the cross-sectional nature of the study.

## 5. Conclusions

In conclusion, this is the first study to identify PMR as a novel and cost-effective biomarker that is associated with multiple forms of dyslipidemia, which constitutes a diagnostic and therapeutic opportunity for CVD and other inflammatory conditions. Future studies should examine the underlying molecular mechanisms governing changes in serum lipids and PMR, the causality between PMR and deranged lipoprotein metabolism, the value of PMR in risk stratification and predicting the development of certain outcomes, and the use of antiplatelet and anti-inflammatory therapeutic interventions in the management of hyperlipidemia and related conditions.

## Figures and Tables

**Table 1:** Characteristics of study subjects.

Characteristic	Normal PMR	High PMR	*p*
Age (years)	39.0 (40.73–41.49)	38.0 (40.07–40.77)	0.052
HCT (%)	42.90 (42.58–42.80)	40.0 (39.80–40.03)	<0.0001
RBC count (×10^6^/μL)	5.39 (5.38–5.41)	5.12 (5.16–5.18)	<0.0001
Hb (g/dL)	14.90 (14.77–14.86)	13.67 (13.62–13.71)	<0.0001
MCH (pg)	27.90 (27.38–27.50)	27.10 (26.36–26.50)	<0.0001
MCHC (g/dL)	34.80 (34.62–34.69)	34.30 (34.12–34.21)	<0.0001
MCV (fL)	79.80 (78.98–79.28)	78.30 (77.10–77.44)	<0.0001
RDW-SD (%)	13.90 (14.30–14.38)	14.10 (14.66–14.76)	<0.0001
ESR (mm/h)	15.0 (36.63–38.25)	10.0 (12.95–13.69)	<0.0001
WBC count (×10^6^/μL)	6.44 (6.61–6.70)	5.18 (5.37–5.45)	<0.0001
FBG (mg/dL)	96.0 (107.3–109.2)	93.0 (101.5–103.2)	<0.0001
HbA_1C_	5.60 (6.09–6.26)	5.40 (5.83–5.94)	<0.0001
Potassium (mEq/L)	4.40 (4.42–4.45)	4.39 (4.38–4.41)	0.0002
Calcium (mg/dL)	9.60 (9.61–9.63)	9.60 (9.61–9.64)	0.7534
Chloride (mEq/L)	104.0 (103.9–104.2)	105.0 (104.5–104.8)	<0.0001
Total protein (g/dL)	7.20 (7.19–7.23)	7.27 (7.24–7.28)	0.0007
Albumin (g/dL)	4.20 (4.19–4.22)	4.20 (4.19–4.22)	0.5563
Globulins (g/dL)	3.0 (2.95–2.99)	3.0 (3.0–3.04)	<0.0001
ALT (U/L)	21.0 (25.59–26.56)	17.0 (21.12–21.95)	<0.0001
AST (U/L)	19.0 (21.15–21.73)	18.0 (19.55–20.12)	<0.0001
ALP (U/L)	69.0 (72.12–74.32)	67.0 (69.26–71.16)	0.0002
Creatinine (mg/dL)	0.80 (0.81–0.83)	0.70 (0.72–0.74)	<0.0001
Urea (mg/dL)	25.0 (26.10–26.77)	22.0 (23.04–23.61)	<0.0001
Uric acid (mg/dL)	5.57 (5.54–5.61)	4.90 (4.98–5.10)	<0.0001
TSH (mIU/L)	1.80 (2.38–2.53)	1.78 (2.41–2.60)	0.2649
Free T_4_ (ng/dL)	1.0 (1.01–1.02)	1.0 (1.01–1.02)	0.4570
Testosterone (ng/dL)	4.58 (4.77–5.0)	4.75 (4.85–5.20)	0.1056
PSA (ng/mL)	0.67 (0.97–1.17)	0.66 (0.70–1.50)	0.2770
CRP (mg/L)	0.33 (0.63–0.72)	0.33 (0.56–0.63)	0.7670
Ferritin (ng/mL)	39.34 (66.63–78.20)	22.28 (41.73–48.52)	<0.0001
25-OH-D_3_ (nmol/L)	14.20 (16.44–16.89)	13.50 (16.28–16.76)	<0.0001

Results are shown as medians ± 95% CI. HCT, hematocrit; RBC, red blood cell; Hb, hemoglobin; MCH, mean corpuscular hemoglobin; MCHC, means corpuscular hemoglobin concentration; MCV, mean corpuscular volume; RDW-SD, standard deviation of red cell distribution width; ESR, erythrocyte sedimentation rate; WBC, white blood cell; FBG, fasting blood glucose; ALT, alanine transferase; AST, aspartate transferase; ALP, alkaline phosphatase, TSH, thyroid-stimulating hormone; PSA, prostate-specific antigen.

**Table 2:** Prevalence of dyslipidemia in light of PMR in studied population.

Marker	Prevalence (%)	*p*
TC		
PMR > 588.6	39.87	
PMR < 588.6	41.73	0.0834
LDL		
PMR > 585.3	75.41	0.1534
PMR < 585.3	77.50	
HDL		
PMR < 586.4	20.43	
PMR > 586.4	32.22	<0.0001
TG		
PMR < 569.3	27.42	
PMR > 569.3	19.33	<0.0001
TC/HDL		
PMR < 563.9	12.84	
PMR > 563.9	8.58	<0.0001
LDL/HDL		
PMR < 596.9	59.97	
PMR > 596.9	51.86	<0.0001
TG/HDL		
PMR < 591.1	63.33	
PMR > 591.1	49.93	<0.0001

**Table 3:** Risk assessment of dyslipidemia in light of PMR.

Parameter	PR	95% CI	*p*	OR	95% CI	*p*
High TC	1.0466	1.0059–1.0889	0.0245	1.0799	1.0100–1.1547	0.0244
High LDL	1.0278	1.0092–1.0468	0.0033	1.1236	1.0397–1.2144	0.0032
Low HDL	1.5771	1.4900–1.6692	<0.0001	1.8514	1.7157–1.9977	<0.0001
High TG	0.7051	0.6638–0.7491	<0.0001	0.6345	0.5865–0.6864	<0.0001
High TC/HDL	0.6690	0.6074–0.7368	<0.0001	0.6379	0.5726–0.7106	<0.0001
High LDL/HDL	0.8647	0.8396–0.8905	<0.0001	0.7189	0.6726–0.7684	<0.0001
High TG/HDL	0.7886	0.7658–0.8120	<0.0001	0.5777	0.5401–0.6178	<0.0001

## Data Availability

Data are available from the corresponding author upon reasonable request, and with permission of Al Borg Diagnostics.
